# Clinical value of epidermal growth factor receptor mutation testing in peripheral blood of patients with non-small cell lung cancer

**DOI:** 10.12669/pjms.40.11.9564

**Published:** 2024-12

**Authors:** Xiaomeng Zheng, Chengbi Tong, Xiaodong Li, Yanan Meng, Xiaodan Liu

**Affiliations:** 1 Xiaomeng Zheng Clinical Laboratory, Affiliated Hospital of Hebei University, Baoding 071000, Hebei, China; 2 Chengbi Tong Clinical Laboratory, Affiliated Hospital of Hebei University, Baoding 071000, Hebei, China; 3 Xiaodong Li Department of Nuclear Medicine, Affiliated Hospital of Hebei University, Baoding 071000, Hebei, China; 4 Yanan Meng Clinical Laboratory, Affiliated Hospital of Hebei University, Baoding 071000, Hebei, China; 5 Xiaodan Liu Clinical Laboratory, Affiliated Hospital of Hebei University, Baoding 071000, Hebei, China

**Keywords:** Non-small cell lung cancer, Peripheral blood, Gene mutation, Epidermal growth Factor receptor, Prognosis

## Abstract

**Objective:**

To investigate the clinical value of EGFR mutation testing in peripheral blood of patients with non-small cell lung cancer.

**Method:**

This was a retrospective study. A total of 89 patients with non-small cell lung cancer (NSCLC) admitted to Affiliated Hospital of Hebei University between June 2019 and May 2023 were selected, and divided into the wild-type group and the mutant group according to the results of EGFR mutation testing in peripheral blood. Clinic pathological data of patients were collected and compared between the two groups, the correlation between EGFR mutation and the prognosis was analyzed.

**Result:**

No statistically significant difference in the mutation rate of EGFR gene was observed between peripheral blood ctDNA and tumor tissue (p=0.879). EGFR mutation in peripheral blood ctDNA was not significantly correlated with sex, age, ethnicity and ECOG score (p>0.05). The EGFR mutation rate was increased in patients with adenocarcinoma compared with that in patient with squamous carcinoma, and in non-smoking patients compared with that in smoking patients, and the differences were statistically significant (p<0.05). The 1-, 2-, and 3-year disease-free survival (DFS) rates were significantly increased in the mutant group compared with those in the wild-type group, respectively (p<0.05).

**Conclusion:**

EGFR mutation in peripheral blood and tumor tissues is highly consistent in NSCLC patients. The EGFR mutation rate is higher in adenocarcinoma and non-smoking patients, and the prognosis is better in patients with EGFR mutations.

## INTRODUCTION

Lung cancer is one of the most common malignant tumors and threatens human health worldwide in recent years. Non-small cell lung cancer (NSCLC) is the main subtype of lung cancer, accounting for about 80% of lung cancer cases.^1^ Early diagnosis is crucial for the treatment and prognosis of NSCLC patients.^2^ Epidermal growth factor receptor (EGFR) is one of the important factors responsible for the development and progression of NSCLC. EGFR mutation is one of the most common pathogenic mutations in NSCLC, with a high incidence especially in Asian population.^3^ EGFR mutation testing in peripheral blood is non-invasive, simple and fast, and reproducible. The progress of molecular biology and high-throughput sequencing technology makes EGFR mutation testing in peripheral blood possible.^4^ On this basis, patients with NSCLC were selected to explore the clinical value of EGFR mutation testing in the peripheral blood of patients with NSCLC.

## METHODS

This was a retrospective study. Eighty-nine patients with Non-small cell lung cancer (NSCLC) admitted to Affiliated Hospital of Hebei University between June 2019 and May 2023 were selected. They were then divided into the wild-type group and the mutant group according to the results of EGFR mutation testing in peripheral blood. There were 50 males and 39 females, with a mean age of 58.37±7.02 years old (range: 48-75 years). 34 patients had a smoking history and 84 patients were Han Chinese. There were 70 cases of adenocarcinoma and 19 cases of squamous carcinoma.

### Ethics Approval:

The study was approved by the Institutional Ethics Committee of Affiliated Hospital of Hebei University (No.: HDFYLL-KY-2022-020; Date: December 30, 2022), and written informed consent was obtained from all participants.

### Inclusion criteria:

- Diagnosed with NSCLC by pathological examination.
- With at least one evaluable tumor lesion.
- With an age of ≥18 years old.
- Signed the informed consent.

### Exclusion criteria:

- With incomplete clinical data.
- Complicated with other malignant tumors.
- With heart, liver, kidney and other organ dysfunction.
- Complicated with mental disorders.

### Methods

### Sample collection:

All pathological specimens were fixed in 4% neutral formaldehyde solution, embedded in paraffin, and sliced into sections. Sections containing >80% tumor cells were used for test. Blood samples were collected in all cases under fasting condition in the morning. Peripheral blood mononuclear cells were immediately sent to the laboratory for isolation and stored at 4°C.

### DNA extraction:

10 sections with a thickness of 5μm were collected, and genomic DNA was extracted using a DNA extraction kit. Purity and concentration of the DNA obtained were measured using a NanoDrop1000 spectrophotometer.

### Detection of EGFR mutation:

Mutations in human EGFR gene were detected by quantitative fluorescent PCR.

### Statistical Analysis:

SPSS22.0 was used for statistical analysis. Measurement data with normal distribution were presented as (*χ̅*±S), and independent sample t-test was used for comparison between groups. The confidence interval was 95%, categorical data were presented as n (%), and χ^2^ test was used for comparison between groups. Differences with a p<0.05 were considered statistically significant.

## RESULTS

Among the 89 patients, wild-type EGFR gene was detected in 52 patients and mutant EGFR gene was detected in 37 patients in their peripheral blood ctDNA, including 18 cases of exon 19 deletion, 14 cases of exon 21 L858R missense mutation, and five cases of exon 20 T790M mutation. There were 38 patients (42.70%) harboring mutant EGFR gene in their tumor tissues, including 16 cases of exon 19 deletion, 18 cases of exon 21 L858R missense mutation, and 4 cases of exon 20 T790M mutation. No statistically significant difference in the EGFR mutation rate was observed between peripheral blood ctDNA and tumor tissue (p=0.879). Table-I and Table-II.

**Table-I:** EGFR mutations in the peripheral blood ctDNA and tumor tissues.

Peripheral blood ctDNA				Tumor tissue			

Wild-type	Mutant			Wild-type	Mutant		

	Exon 19 deletion	Exon 21 L858R missense mutation	Exon 20T790Mmutaion		Exon 19 deletion	Exon 21 L858R missense mutation	Exon 20T790Mmutaion
52	18	14	5	51	16	18	4

**Table-II:** Comparison of EGFR mutations between peripheral blood ctDNA and tumor tissues.

EGFR mutations in tumor tissue	EGFR mutations in peripheral blood ctDNA		Total

	Positive	Negative	
Positive	51	0	51
Negative	1	37	38

Total	52	37	89

EGFR mutation in peripheral blood ctDNA was not significantly correlation with sex, age, ethnicity, and ECOG score (p>0.05). The EGFR mutation rate was increased in patients with adenocarcinoma compared with that in patient with squamous carcinoma, and in non-smoking patients compared with that in smoking patients, and the differences were statistically significant (p<0.05) (Table-III).

**Table-III:** Relationship between EGFR mutation in peripheral blood ctDNA and the clinical features of patients with NSCLC.

Features	n	The mutant group(n=37)	The wild-type group(n=52)	χ^2^	P
Sex				0.009	0.923
Male	50	21(42)	29(58)		
Female	39	16(41.03)	23(58.97)		
Age(years)				0.039	0.843
≥60	47	20(42.55)	27(57.45)		
<60	42	17(40.48)	25(59.52)		
Smoking history				9.974	0.002
Yes	34	7(20.59)	27(79.41)		
No	55	30(54.55)	25(45.45)		
Ethnicity				0.005	0.941
Han	84	35(41.67)	49(58.33)		
Non-han	5	2(40)	3(60)		
Pathological type				9.583	0.002
Adenocarcinoma	70	35(50)	35(50)		
Squamous carcinoma	19	2(10.53)	17(89.47)		
ECOG score(points)				0.001	0.979
0-1	60	25(41.67)	35(58.33)		
≥2	29	12(41.38)	17(58.62)		

Binary logistic regression analysis was conducted with EGFR mutation in peripheral blood ctDNA as the dependent variable (0=yes, 1=no), and smoking history and pathological type as the independent variables. It was found that pathological type (adenocarcinoma) was a risk factor for EGFR mutation in peripheral blood ctDNA (p<0.05), and smoking history was a protective factor for EGFR mutation in peripheral blood ctDNA (p<0.05) (Table-IV).

**Table-IV:** Multivariate logistic regression analysis.

Variables	B	S.E.	Wald	df	P	OR	95% CI	

							Lower limit	Upper limit
Smoking history	-1.838	0.538	11.681	1	0.001	0.159	0.055	0.457
pathological type (adenocarcinoma)	2.508	0.817	9.431	1	0.002	12.286	2.478	60.911
Constants	0.458	1.182	0.150	1	0.699	1.580		

All patients were followed up for three years. Given the limited number of deaths, DFS rate was used in this study as the primary outcome measure. The 1-, 2-, and 3-year DFS rates were significantly increased in the mutant group compared with those in the wild-type group, respectively (p<0.05) (Table-V).

**Table-V:** The relationship between EGFR mutation and the prognosis of NSCLC.

Groups	1-year DFS rate	2-year DFS rate	3-year DFS rate
The mutant group (n=37)	35(94.59)	23(62.16)	17(45.95)
The wild-type group (n=52)	41(78.85)	21(40.38)	13(25)
*χ* ^2^	4.298	4.102	4.244
*P*	0.038	0.043	0.039

## DISCUSSION

In the present study, EGFR mutations were detected in patients, and no significant difference was found in the EGFR mutation rate between peripheral blood ctDNA and tumor tissues (p=0.879). EGFR mutation is one of the common driver mutations in patients with NSCLC. CtDNA can be used to detect tumor-related gene mutations by using peripheral blood samples. Studies have found that the release of ctDNA is affected by various factors, including tumor size, blood supply, and tumor secretions. Therefore, the amount of ctDNA released may vary at different time points and under different conditions, and consequently EGFR mutations may not be detected in ctDNA in some cases, even when EGFR mutations are identified in tumor tissues.^5^-^7^ The sensitivity of ctDNA testing is limited, and low-frequency EGFR mutations may not be detected, although modern ctDNA testing techniques are already quite sensitive. Therefore, the results of ctDNA testing may be negative even if EGFR mutations are present in tumor tissues.^8^ Heterogeneity is also a possible contributing factor. Different genetic variations may occur in different regions of the tumor tissues, and ctDNA only represents part of the tumor. Therefore, in some cases, EGFR mutations found in ctDNA may not be completely consistent with those in tumor tissues.

Non-small cell lung cancer(NSCLC) is the most common type of lung cancer, accounting for about 80% of lung cancer cases. The invasion and metastasis of NSCLC is high, although the progression of this disease is slow relative to other types of lung cancer.^9^ EGFR mutation refers to the abnormal variations of EGFR gene. EGFR is a protein receptor that locates on the surface of cells and participates in the regulation of cell growth and division.^10^ Mutations in EGFR gene may lead to abnormal activation of the EGFR signaling pathway, which in turn promotes the proliferation and survival of tumor cells. Therefore, EGFR mutation testing is of great importance in the diagnosis and treatment of NSCLC.^11^-^14^ After detection of EGFR mutations in patients, individualized treatment can be developed according to their mutation types to improve the treatment efficacy and survival rate of patients.

There was no significant correlation between EGFR mutation in peripheral blood ctDNA and sex, age, ethnicity and ECOG score (p>0.05), and the EGFR mutation rate was increased in patients with adenocarcinoma compared with that in patients with squamous carcinoma, and in non-smoking patients compared with that in smoking patients, with statistically significant differences(p<0.05). This possibly be explained by the differences in pathological features and mechanism of progression between adenocarcinoma and squamous carcinoma.^15^ EGFR mutations are more prevalent in adenocarcinomas than in squamous carcinoma, which may be related to the differences in cell type and differentiation status between the two types of NSCLC.

The relationship between smoking and EGFR mutation has been extensively studied.^16^-^18^ Although smoking is associated with lung cancer in many cases, it can inhibit the incidence of EGFR mutations. Therefore, the EGFR mutation rate is relatively low in patients who smoke. Smoking may reduce the probability of EGFR mutations by inducing mutations in other carcinogenic genes or affecting the DNA repair mechanism in cells. In contrast, the incidence of EGFR mutations is higher in non-smoking patients^19^, and this may be contributed by the differences in genetic susceptibility, environmental exposure, and other carcinogenic mechanisms between smoking and non-smoking individuals. In patients with no smoking history, EGFR mutations may be more of a driver of lung cancer.

The 1-, 2-, and 3-year DFS rates were significantly increased in the mutant group compared with those in the wild-type group(p<0.05). Studies have shown that mutations in some genes, such as EGFR, and ALK, are drivers of NSCLC progression. These mutations can promote abnormal proliferation and survival of tumor cells, and potentially make the disease more sensitive to some treatments. Therefore, patients in the mutant group generally response better with an improved DFS rate when receiving related targeted therapies. These targeted therapies can precisely target specific gene mutations to block tumor cell growth signals, and provide a longer DFS.^20^

In contrast, absence of these specific genetic mutations in patients of the wild-type group may lead to an increased resistance to targeted treatment and subsequently a decreased DFS rate. The differences may be significant in the 1-, 2-, and 3-year DFS rates and reduce over time due to various factors such as the development of treatment resistance. Therefore, targeted therapies in patients with NSCLC are still challenged in terms of long-term survival.

### Limitations:

It includes a small number of patients who were included and no long-term follow-up was conducted. In future more patients should be included and follow-up time should be increased to further validate the findings of this study.

## CONCLUSIONS

Epidermal growth factor receptor mutation in peripheral blood and tumor tissues is highly consistent in patients with NSCLC. EGFR mutation rate is higher in adenocarcinoma and non-smoking patients, and the prognosis is better in patients with EGFR mutations.

### Authors’ Contributions:

**XZ:** Carried out the studies, data collection, and drafted the manuscript.

**CT, XL, YM** and **XLiu:** Performed the statistical analysis and participated in its design. Critical Review.

All authors have read, approved the final manuscript and are responsible and accountable for the accuracy or integrity of the work.
